# Compact, modular and in-plane AOSLO for high-resolution retinal imaging

**DOI:** 10.1364/BOE.9.004275

**Published:** 2018-08-15

**Authors:** Laura K. Young, Timothy J. Morris, Christopher D. Saunter, Hannah E. Smithson

**Affiliations:** 1 Department of Experimental Psychology, University of Oxford, 15 Parks Road, Oxford OX1 3PH, UK; 2 Department of Physics, Durham University, South Road, Durham DH1 3LE, UK

**Keywords:** (170.4460) Ophthalmic optics and devices, (170.5755) Retina scanning, (110.1080) Active or adaptive optics, (180.5810) Scanning microscopy, (180.1790) Confocal microscopy

## Abstract

The adaptive optics scanning laser ophthalmoscope (AOSLO) was first developed in 2002 and since then the technology has been adopted in several laboratories around the world, for both clinical and psychophysical research. There have been a few major design implementations of the AOSLO. The first used on-axis tilted spherical mirrors in a planar arrangement, and the second minimized the build up of astigmatism present in the first design by using a non-planar arrangement. Other designs have avoided astigmatism by using custom-made toroidal mirrors or by using lenses on-axis, rather than mirrors.

We present a new design implementation for an AOSLO that maintains a planar optical alignment without the build up astigmatism using compact, reconfigurable modules based on an Offner relay system. We additionally use an off-the-shelf digital oscilloscope for data capture and custom-written Python code for generating and analyzing the retinal images. This design results in a compact system that is simple to align and, being composed of modular relays, has the potential for additional components to be added. We show that this system maintains diffraction-limited image quality across the field of view and that cones are clearly resolved in the central retina. The modular relay design is generally applicable to any system requiring one or more components in the pupil conjugate plane. This is likely to be useful for any point-scanned system, such as a standard scanning laser ophthalmoscope or non-ophthalmic confocal imaging system.

## 1. Introduction

Retinal imaging has long been a useful diagnostic tool in ophthalmology and the invention of the scanning laser ophthalmoscope (SLO) [1–3] marked an important step in improving the resolution of such images. This system is a scanning confocal microscope in which the participant’s eye serves as the objective lens. Optical aberrations in the eye degrade the quality of the focused beam on the first pass and further degrade it as the light is reflected back out of the eye. The confocal pinhole acts to spatially filter the reflected light to improve the effective resolution, but with a reduction in the amount of light reaching the detector. A larger pinhole can be used to improve the throughput but at the expense of allowing the optical aberrations to impact upon the image quality.

The first use of adaptive optics (AO) to improve the resolution and throughput of a scanning laser ophthalmoscope by producing a high-quality focus at the pinhole was achieved by Roorda *et al*. in 2002 [4], making imaging of individual cones close to the fovea possible. In the time since, the adaptive optics scanning laser ophthalmoscope (AOSLO) has become important for studying the anatomy and morphology of the retinal components at a cellular scale [5, 6]. Images obtained with an AOSLO give *in vivo* structural information such as the arrangement and density of the photoreceptors as well as the size, composition and arrangement of the blood vessels [7]. Further developments have opened up the possibility of studying other retinal structures such as inner segments of cones, imaged via split-detection [8], the retinal pigment epithelium, imaged via dark-field techniques [9] and retinal ganglion cells imaged via offset-aperture imaging [10]. AOSLO imaging is increasingly being used to study the function of the retina, such as using single-cell stimulation to understand the neural wiring in the retina [11], monitoring blood flow [12] and metabolic activity [13], and analyzing motion distortions within the images for eye tracking at high spatial and temporal resolution [14–16].

To date, reflective AOSLOs used around the world have been built broadly to one of three designs. The first generation AOSLO [4,17] used tilted spherical mirrors on-axis to create a series of 4-f relays, re-imaging the pupil of the eye onto horizontal and vertical scanners as well as onto a deformable mirror (DM). The use of mirrors, rather than lenses, eliminates back reflections that would otherwise contaminate the (wavefront sensor) WFS image, avoids chromatic aberration, and gives a higher throughput. However, using spherical mirrors in this way introduces astigmatism into the wavefront and this accumulates with each reflection from a spherical mirror. Later, Dubra and Sulai [18] and Merino *et al*. [19] developed a second-generation AOSLO that balanced astigmatism in one axis with astigmatism in the perpendicular axis by using an out-of-plane design in which the optical path is not contained in a plane parallel to the optical bench. These systems were more compact and removed the need for an astigmatic correction lens by reducing the astigmatic errors to a simple focus term. This led to a diffraction-limited field of view of 1° at a wavelength of 450 nm. In 2013 Liu *et al*. [20] developed an in-plane AO-OCT optical design that avoided the introduction of astigmatism by using toroidal, rather than spherical mirrors. This system achieves diffraction-limited performance within ±1.8° at 800 nm. However, toroidal mirrors require custom specification and manufacture, and are more expensive than the spherical mirrors used here.

Although astigmatism can be avoided by using lenses rather than spherical mirrors, lenses have traditionally been avoided when designing AOSLOs. Back reflections from lenses can affect image quality and WFS performance and can be comparable in intensity to the signal from the retina. In 2012, Felberer *et al*. [21] developed a lens-based system that uses a pair of quarter-wave plates to remove back reflections from the optics. This allowed a more compact system to be developed with a larger theoretical diffraction-limited field of view (good image quality is achieved with a 4° field of view at a wavelength of 840 nm). However, lens-based systems are still limited due to chromatic aberration and wavelength-dependent polarization effects. Additionally, this lens-based system relies on the polarization of light being unaltered by the sample, which may not be the case when light is scattered (e.g. in retinal pigment epithelium cell imaging) or when imaging fluorescence emission.

One goal of the lens-based AOSLO design is to achieve diffraction-limited imaging using a compact instrument. Two issues in the commercialization of AO retinal imaging techniques are cost and the space required for such instruments. Efforts have been made to make AO-assisted instruments such as the AOSLO [22–24], the AO-OCT [22,23,25] and the AO fundus camera [25] more compact and therefore easier to adopt in the clinic. Such efforts indicate that the development of more compact systems is beneficial for the wider use of AO retinal imaging technologies. The system presented here uses a reflective, rather than refractive, design that carries additional benefits where broadband or multi-wavelength light sources are used.

We present a new compact AOSLO design that is in-plane, simplifying the construction and alignment, and does not suffer from the build-up of astigmatism. Our system is composed of three configurable fully-reflective pupil relay modules, one for each scanning mirror and one for the DM. Such a design could in principle also be used for other types of scanned light microscopy, where pupil conjugate planes must be re-imaged. The system is also compact: the AOSLO relay has a footprint of just 350 × 300 mm and the entire system fits within an area of approximately 1.0 × 0.5 m. Rather than using a frame-grabber we use a digital oscilloscope with custom software to reconstruct the raw images. This allows access to the raw signals and so increases the flexibility of data capture (e.g. programming the pixel clock) without the use of a field programmable gate array (FPGA). The use of a digital oscilloscope has advantages over a FPGA since it does not require specialist knowledge and custom development in a hardware description language. The system is built entirely using off-the-shelf optical and electronic components with custom code, written in the Python programming language [26].

## 2. Methods

### 2.1. Optical design

**Fig. 1:**
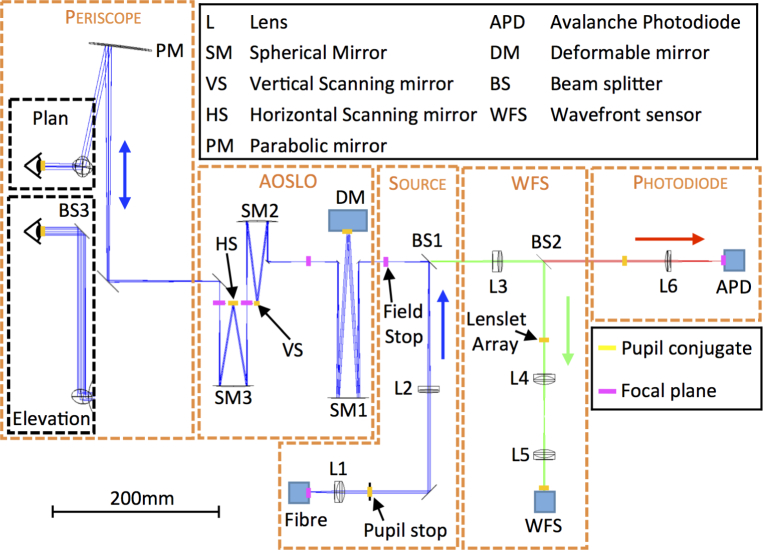
System optical layout showing the five functional modules within the AOSLO system. The source module (Sec. 2.1.1) relays the superluminescent diode (Fibre) via an 8% reflection from BS1 to the AOSLO module (Sec. 2.1.2), where the scanning and wavefront compensation takes place. The scanned, wavefront-compensated light is delivered to the eye via the periscope module (Sec. 2.1.3) and light returning from the eye passes back through the AOSLO module. BS1 transmits 92% of the returned light into the next two modules, of which 8% is reflected into the WFS module (Sec. 2.1.4), where the wavefront is sensed. The remaining 92% of the light is transmitted by BS1 is to the photodiode module, where the light is detected. Light propagation direction within modules is indicated by the colored arrows.

The optical layout of the system is shown in Fig. 1 and has been separated into five functional modules. Here we describe the optical design of each module, finally providing an analysis of end-to-end system performance. System optical performance was modeled using the Zemax optical design software. Unless stated otherwise, for the purposes of image scaling throughout this design we assume a paraxial model eye with a pupil diameter of 7 mm and a focal length of 17 mm.

#### 2.1.1. Source module

The light source used within the AOSLO is a fiber-coupled 850 nm (50 nm FWHM) superluminescent diode (BLMS-mini-351-HP3-SM-OI, SuperLum). The output fiber (P5-780A-PCAPC-1, Thorlabs) has a mode-field diameter of 5 *µ*m with a numerical aperture of 0.12. A lens of focal length 40 mm (AC254-40-B-ML, Thorlabs) is used to collimate the light from the fiber and therefore the projected image of the fiber at the model retina has a nominal diameter of 2.1 *µ*m.

**Fig. 2:**
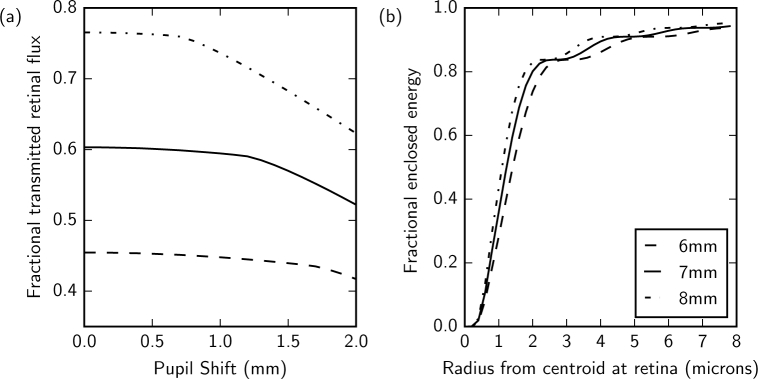
(a) The fraction of transmitted flux at the pupil compared to flux after the source aperture with offset of the eye pupil from the center of the Gaussian intensity profile for 6, 7 and 8 mm diameter eye pupils. The change in gradient at large pupil offsets for 7 and 8 mm diameter pupils corresponds to the pupil moving outside the 9.4 mm diameter illuminated area of the eye. (b) Modeled on-axis fractional enclosed energy for the 5 *µ*m diameter source fiber projected onto the retina for eye pupil diameters of 6 to 8 mm at 850 nm.

After the collimating lens, a circular aperture conjugated to the eye pupil is used to define a beam of 4 mm in diameter. This aperture corresponds to a projected diameter of 9.4 mm at the eye pupil that has a Gaussian intensity profile with a 1/*e*^2^ diameter of 21.8 mm. Motion of the pupil within this intensity profile causes a variation in flux at the retina, as modeled in Fig. 2. For a 7 mm diameter pupil, up to 1.2 mm of pupil motion is possible before vignetting of the pupil occurs. A bite-bar and dental impression is used during imaging and we can reasonably expect the pupil position to be stable to within 1 mm, resulting in a flux variation of up to 1.5%.

The final elements in the source module are a 200 mm focal length lens (L2: AC254-200-B, Thorlabs) creating a telecentric output beam with an f-ratio of 49.4. A 92/8 beamsplitter (BS1: CP1-BP108, Thorlabs) reflects 8% of the light from the fiber into the system. A variable aperture field stop is placed at the focus after the beamsplitter to reject any stray and scattered light returned through the system, as well as provide an alignment reference target. The diameter of this aperture is much larger than the diffraction limit and therefore does not impact the measured wavefront.

The AO system only corrects for aberrations present between the retina and the WFS. Non-common path optical aberrations between the WFS and the pinhole can be calibrated (see section 3.1) however aberrations within the source optical path, which is not part of the double pass path, cannot be compensated. Residual aberrations in the source path increase the size of the illuminated patch at the retina, ultimately lowering flux through the confocal pinhole and increasing the size of the WFS spots. Differential aberrations between these optical paths have been estimated from the design based on alignment accuracy and element manufacturing errors described in Table 1. A Monte-Carlo tolerance analysis of the expected system performance after alignment predicted a FWHM of 2.4±0.1 *µ*m for a 7 mm diameter pupil (compared to the diffraction-limited FWHM of 2.1 *µ*m). Figure 2 shows the predicted encircled energy radius at the retina for 6, 7 and 8 mm pupil diameters with the 5 *µ*m diameter source fiber. For the 7 mm diameter pupil, 80% of the light incident on the retina is contained within a 2 *µ*m radius circle.

**Table 1:** Optical element alignment and manufacturing errors used within the tolerance analysis.

Error term	Magnitude	Comment
Lens position along optical axis	±0.5 mm	
Lens translation	±0.25 mm	Perpendicular to optical axis.
Lens tilts	±0.5 deg	
Lens thickness	±0.1 mm	
Refractive index errors	±0.001	
Surface shape errors	*λ*/10	Restricted to first 15 Zernike aberrations.
Surface curvature errors	±1%	Percentage of surface radius of curvature.

#### 2.1.2. AOSLO relay

**Fig. 3:**
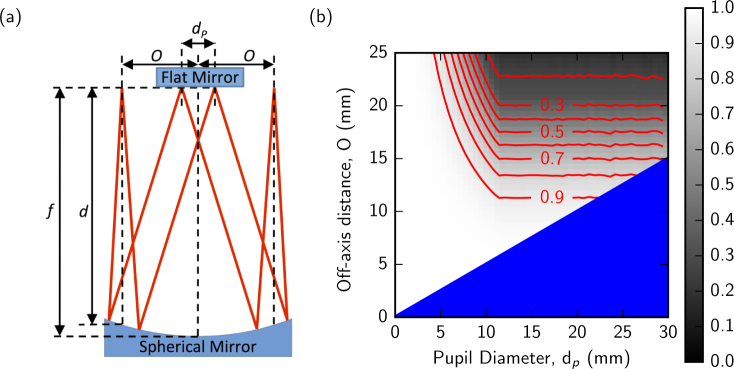
(a) Schematic of the light path through a spherical relay showing the pupil diameter, *d_p_*, and the offset distance from the optical axis of the spherical mirror, *O*. (b) The Strehl ratio of a re-imaged 850 nm point source using a 200 mm focal length spherical relay varying the pupil diameter and offset distance. The blue area indicates the region that is physically impossible since the off-axis distance cannot be less than half of the diameter of the pupil.

The AOSLO relay contains a series of reflective spherical mirror modules, creating pupil-conjugate planes at the locations of the DM (3.5 *µ*m stroke MultiDM, Boston Micromachines) and each of the scanning mirrors (PLD-XYG, SC-30 16 kHz fast scan and Electro-Optical Products Corporation). Each pupil conjugate module is based upon the Offner relay [28], which re-images an input focal plane with 1:1 magnification using two concentric spherical mirrors. Here we replace the convex mirror of the true Offner relay with a flat mirror (either the DM or either of the scanning mirrors). This reduces the optical performance of the system compared to the true Offner relay. However diffraction-limited performance can still be achieved if the combination of operating wavelength, pupil diameter and off-axis distance remains within a limited parameter space. Figure 3 shows the output Strehl ratio (with a Strehl ratio of 1 describing zero wavefront error) through a 200 mm focal length spherical relay as the pupil diameter and off-axis distance are varied. For larger pupil diameters, the reduction in Strehl ratio becomes significant, but the effect can be minimized by reducing the off-axis distance.

The parameters of each relay are constrained by the size of each of the components that must be relayed. Wavefront compensation is achieved using a square geometry 140 actuator, 400 *µ*m pitch DM (MultiDM, Boston Micromachines). The active area of the DM is 4.2 mm diameter, requiring a 200 mm focal length spherical mirror (SM1: CM508-200-E02, Thorlabs) relay to give the correct magnification at the eye pupil. As the DM enclosure is wider than the spherical mirror relay, two D-shaped 5 mm diameter fold mirrors (Thorlabs PFD05-03-P01) are used to direct the input and output beams away from it. The double-pass path through the protective window of the DM does not impact optical performance at a level that would be observable in the complete system. To ensure collimation after reflection from the spherical mirror, the axial position of the spherical mirror must be adjusted compared to the nominal focal length to take into account the surface curvature and off-axis distance. The corrected distance between mirror and focal plane, *d* is given by

$$d=\sqrt{f^{2}-O^{2}}$$ (1)

where *f* is the focal length of the spherical mirror, and *O* is the off-axis distance as defined in Fig. 3. For the 12.5 mm off-axis distance used in the DM relay, *d* = 199.6 mm.

**Fig. 4:**
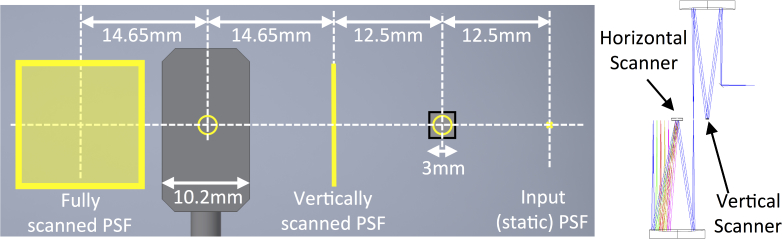
The mirror configuration in the plane of the scanning mirrors from input unscanned PSF to the output scanned focal plane. Beam diameters at all intermediate planes are shown in yellow and a plan view of the optical path through scanning mirrors is shown on the right.

The pupil diameter at both the fast (vertical) and slow (horizontal) scanning mirrors is 2.1 mm, produced using 100 mm focal length spherical mirror (SM2/SM3: Thorlabs CM508-100-E02) relays. The optical layout of the scanning system (Fig. 4) is defined by the requirement to avoid vignetting of the scanned output beam by the final spherical mirror. The scanning system in this configuration has maximum scan angle of ±2deg, corresponding a square section of the retina approximately 0.6 mm in width (dependent upon the optical properties of the eye).

**Fig. 5:**
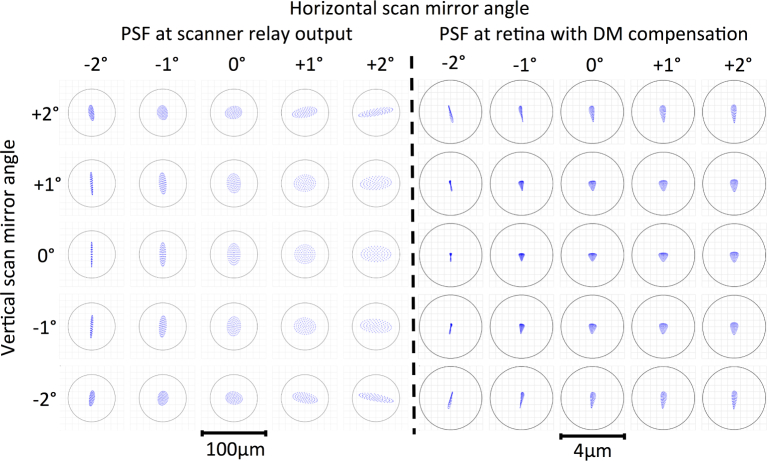
Ray-trace through scanning relay (left) and at the retina of the model eye (right) showing PSF every degree over the ±2 deg scanning mirror range compared to the diffraction-limited Airy diameter at 850 nm (black circles). Common aberrations present at the retina have been compensated using the DM. The −2 deg horizontal scan angle is the field point closest to the slow scanning (horizontal) mirror.

Figure 5 shows the ray-trace for field points every degree within the ±2 deg scan range. At each field point, the ray-trace remains within the Airy diameter and therefore the system remains diffraction-limited over this scanning range at this wavelength. Note that the diffraction limit referred to within Fig. 5 describes the diffraction limit of the maximum pupil diameter (fully illuminated DM) and does not take into account the smaller pupil diameter of the eye. The scanned field has a mean image scale of 0.278 mm per degree of scan at the model-eye retinal plane. Optical aberrations can lead to some anamorphism in the reconstructed image. The optical design predicts a maximum deviation of the position of the PSF from the expected position on the retina of 2.4 *µ*m. Mean deviation is 0.71 *µ*m with a standard deviation of 0.47 *µ*m. We correct for this *a posteriori* through calibration using a grid target placed in the scanned focal plane (e.g. Edmund Optics 62-209).

#### 2.1.3. Periscope

The functions of the periscope are to create a collimated beam with an accessible DM/scanning mirror conjugate plane at which the participant’s pupil can be placed, and to include a beamsplitter that separates the 850 nm AOSLO light from visible wavelengths. This allows the participant to be shown a distant background fixation target during experiments. One advantage of the in-plane AOSLO relay design is that a 100 mm height periscope can raise the beamsplitter (BS3: DMSP805L, Thorlabs) above the main optical system and provide a wide field of view for background fixation targets and other psychophysical stimuli.

Initially a refractive relay was designed, however back reflections from the long focal length lens were significantly brighter than the signal backscattered from the retina. A reflective relay was developed for the final system using an off-axis segment of a 444 mm focal length parabolic mirror (PM: Edmund Optics 32-064-566). This provides a 9.35 mm diameter collimated beam at the pupil positioned 444 mm from the parabolic mirror. For optimal alignment of an off-axis parabolic mirror, the off-axis distance and mirror tilt angle, *θ*, are related by

θ=arctan(O/f) (2)

For the 76.2 mm diameter parabolic mirror the maximum off-axis distance that reflects the scanned field without vignetting is 25 mm, defining a 3.22 deg tilt angle. This shallow angle does not provide sufficient space to separate input and output beams, therefore the angle must be increased to allow for the periscope optomechanics. The DM can be used to compensate for any optical aberrations that are common across the field at the expense of DM stroke. Including this degree of freedom allows the parabolic mirror tilt angle to be increased to 7 deg at an off-axis distance of 14.4mm. This compensates for the majority of field-dependent aberrations present within the scanning system. Whilst the relay output remains diffraction-limited under these conditions with a residual RMS wavefront error across the full scanned region of better than *λ*/10, the addition of a small astigmatic term on the DM (peak-to-valley amplitude on the DM surface of ±0.11 *µ*m) can also be applied to correct for common aberrations. This corresponds to 6.2% of the DM mechanical stroke and results in a RMS wavefront error across the full scanned region of the retina of better than *λ*/50 at 850 nm.

The limited DM stroke, shallow beam angles and available space envelope around the scanned focal planes makes it difficult to integrate gross focus compensation within the system without an additional optical relay. Ophthalmic trial lenses are therefore used to correct for large focus terms. We have not observed additional back reflections within the system from the use of these lenses placed near the eye pupil. The use of a Badal lens system could also be implemented to provide correction of large focus terms or a low-order DM could be added to the system (e.g. [29]).

#### 2.1.4. Wavefront sensor

Approximately 7.4% of the return flux from the eye is directed towards the WFS after transmission through BS1 and reflection from BS2. The WFS relay optics re-image the 12 × 12 actuator DM surface onto 11 × 11 lenslets of a 300 *µ*m pitch, 5.1 mm focal length lenslet array (18-00211, SUSS micro-optics) using a 150 mm focal length lens (L3: Thorlabs AC254-150-B). This results in each sub-aperture of the WFS corresponding to 0.9 × 0.9 mm at the eye pupil. Optical distortion between the lenslet array and DM is less then 0.1% which ensures the lenslet array remains aligned to the DM in the optimal Fried geometry [30].

The resulting lenslet spot pattern is then re-imaged onto the CCD (Edmund Optics EO-0312M) using a 1:1 refractive relay comprising two matched 40 mm focal length achromatic lenses (L4 and L5: Thorlabs AC254-040-B). Achromatic lenses were used within this design because they provided superior imaging performance across the field compared to singlet lenses of similar focal length. An additional benefit of the refractive WFS relay is that it creates an accessible DM conjugate plane in which the WFS camera can be placed during alignment. This enables accurate conjugation of the lenslet array to the DM plane. The relay also simplifies optomechanical mounting because the lenslet array need not be placed within 5 mm of the detector. The designed WFS centroid response after the relay across a ±2 deg input field angle remains linear to better than 1%.

The FWHM of the optimal retinal PSF (defined in Sec. 2.1.1) within each subaperture is 8.123 *µ*m, or 0.821 pixels. The WFS is not confocal, therefore sensing operates on light backscattered from the retina that is not limited to a single plane. For a scattering depth of ±40 *µ*m, defocus within the WFS increases the PSF FWHM to >1 pixel, avoiding sub-sampling effects within the WFS that can cause non-linearities in WFS response.

#### 2.1.5. Photodiode path

The photodiode path receives the collimated beam after BS2 (84% of the return flux present at the field stop). A 125 mm focal length lens (L6: Thorlabs AC254-125-B) focuses light onto one of a set of pinholes that range in size from 20 to 300 *µ*m in diameter. We can estimate that a 89.9 *µ*m diameter pinhole is required to enclose 50% of the flux from the retina.

### 2.2. Adaptive optics system

Adaptive optics correction is achieved using a microelectromechanical (MEMS) DM (see Sec. 2.1.2) and a custom-made Shack-Hartmann WFS (see Sec. 2.1.4). The WFS subapertures are aligned to the DM in the optimal Fried geometry [30] and the WFS and DM are used in a closed-loop configuration.

Real-time control is achieved using custom code, written in the Python programming language and the NumPy multidimensional array library [26]. Before an imaging session an interaction matrix is generated, mapping DM actuator voltages to spot motion in the WFS using a 2.4 × scale model eye. The control matrix is calculated as the pseudo-inverse of this matrix using a singular value decomposition. The reconditioning value used in the singular value decomposition was selected as that giving the best closed-loop stability on testing.

On each cycle of the closed loop, intensity thresholding is used to define the subapertures that should be included in the calculation of the actuator voltages, allowing for some movement of the eye pupil. The center of mass of the image from each subaperture is calculated, from which the null spot positions are subtracted, to measure the tip and tilt of the wavefront at each subaperture. The global tip and tilt aberrations, given by the average horizontal and vertical centre of mass shifts, are removed. Multiplication of a vector comprising these spot motions with the control matrix yields the necessary changes to the actuator values to restore the null wavefront, with these changes being integrated over time. Where actuators fall just outside the pupil their voltages are set to the average of their nearest neighbours. The loop gain is set automatically using a measure of closed-loop stability that is based on the centroid position variance over two seconds, with a typical gain being 0.3.

System aberrations, including non-common path errors, cause a spread in the area of retina illuminated and a reduction in the amount of light focused through the pinhole, reducing the signal-to-noise ratio (SNR). Such aberrations are compensated using an image optimization protocol. The 2.4 × scale model eye was placed in the system and the residual system aberrations were compensated with the DM using the Nelder-Mead simplex algorithm [27] to find the actuator voltages necessary to optimize the output at the pinhole via one of two methods. The first method used a CCD placed directly in the focal plane of the AOSLO, where the pinhole is normally located, and maximizes the sharpness of the PSF, which we define as

$$\epsilon =\frac{\sum I{\left(x,y\right)}^{2}}{{\left(\sum I(x,y)\right)}^{2}},$$ (3)

where *I*(*x, y*) is the intensity of pixel (*x, y*). The second method used the raw image from the AOSLO collected through the pinhole and minimizes the inverse of the total intensity in the image, maximizing the throughput of the pinhole. The wavefront centroid positions are measured whilst this mirror vector is applied and are used as the null reference positions to which the closed loop attempts to converge.

### 2.3. Data capture

In an AOSLO, retinal images are reconstructed based on the scan position and the signal from the imaging detector. Our system uses an off-the-shelf digital oscilloscope (Picoscope 3403B, Picotech) to record these signals, which are streamed to the control PC in real-time. Other than a 20MHz bandwidth filter included in the oscilloscope, all signal processing is done in software. The maximum data rate of the oscilloscope over USB 3.0 is 125 MSamples/s shared between the three channels that we use. The scanning system has 533 lines (forward and reverse) per frame and a frame rate of 30 Hz. This allows us to capture up to 2605 intensity samples per scan line, 1302 per direction. The desired field of view of the system is 1° across a scan line and, to achieve Nyquist sampling, the pixel scale should be half of the lateral resolution, i.e. 13 arcseconds (1 *µ*m on the retina), giving a minimum number of pixels per scan line of 277 in each direction. We are therefore not limited by the data rate of the oscilloscope and we are able to oversample (and average) by a factor of up to 4.7. The digital oscilloscope has a discrete number of available sampling intervals, we chose a sampling interval of 24 ns, which allowed the highest possible integer oversampling rate (four). To account for the non-integer number of pixels per scan line we perform a 1D interpolation on the raw intensity data before downsampling by a factor of four via averaging.

### 2.4. Image reconstruction

Data from the digital oscilloscope is received as a continuous stream and individual frames are separated using a software trigger that is based on the scan position, which uses hysteresis to improve the triggering accuracy. There is a small time difference between the mechanical motion of the scanner and its monitoring signal that must be accounted for. Therefore, fine tuning of the software trigger is performed in post-processing by comparing the forward and reverse scan intensity data, which should be almost identical except for noise.

The scan pattern of the AOSLO is non-linear and so captured intensity data must be linearized using the scan position data. The horizontal, fast scan is produced by a resonant scanner with a sinusoidal pattern and the vertical, slow scan is produced by a galvanometer with an approximately linear pattern (ignoring the fly-back). To reduce noise on the scan position measurement we produce fits to the data (a sinusoidal fit to the horizontal, fast scan data and a second order polynomial fit to the vertical, slow scan data) and this is used as a look-up table to produce linear spatial sampling.

For display purposes during the imaging session, only coarse triggering and non-integer pixel compensation are performed, allowing a live video to be viewed at close to real-time. Fine-tuning of the software trigger and linearizing the data is performed on a frame-by-frame basis in post processing, which accounts for any changes in the scan pattern during the imaging session. The result of these data processing steps is a pair (forward and reverse scan) of 480 × 330 pixel images of a 1.6 ° × 1 ° patch of the retina. The size of the scan patch is limited by the mechanical range of the scanner and not by image quality since diffraction-limited imaging is theoretically possible over ±2°. Typically, these two images are additionally averaged to further suppress noise, such that each pixel in the final image is effectively an average of eight samples (single forward or reverse frames are oversampled and averaged by a factor of four). We store all the raw data from the oscilloscope allowing us to resample and re-process the images as necessary. The final image can also be composed by interleaving the forward and reverse scan lines and by maintaining an oversampling factor of two, giving a 960 × 660 pixel image of the same patch of retina, in which each pixel is an average of two samples.

### 2.5. Imaging protocol

Imaging and wavefront sensing is carried out using an 850 nm (50 nm FWHM) superluminescent diode (Superlum). The AOSLO imaging path is directed to the eye via reflection from a dichroic filter, allowing visible wavelengths (<800 nm) to pass through. A display is located at the end of the bench 1.75 m from the eye and in a plane that is conjugate to the focal plane of the AOSLO. We generally avoid using cycloplegic eye drops where possible, allowing participants to focus naturally on the display, and the selection of a long imaging wavelength significantly reduces the impact of the illumination on pupil dilation. Participants are positioned on an adjustable bite-bar to maintain eye-position stability and the eye is aligned to the system using the wavefront-sensor image as a guide. Trial lenses are used to correct for large refractive errors, or to induce a focus shift for viewing different retinal layers.

Retinal locations are targeted by directing the participant’s fixation via a target on the display, assuming foveal fixation in normal eyes. Where appropriate, in patients with diseased retina, we adjust the relative position of the target according to their preferred retinal locus and use features in their fundus and OCT images to check accuracy. Light collection is performed using either an avalanche photodiode (APD410A, Thorlabs) or photomultiplier tube (H7422-50, Hamamatsu), depending on the sensitivity required. The minimum voltage range on the digital oscilloscope is ±50 mV, so when using the avalanche photodiode with very low signal levels an additional (10 times) voltage amplifier is required.

## 3. Results and discussion

### 3.1. System aberrations

**Fig. 6:**
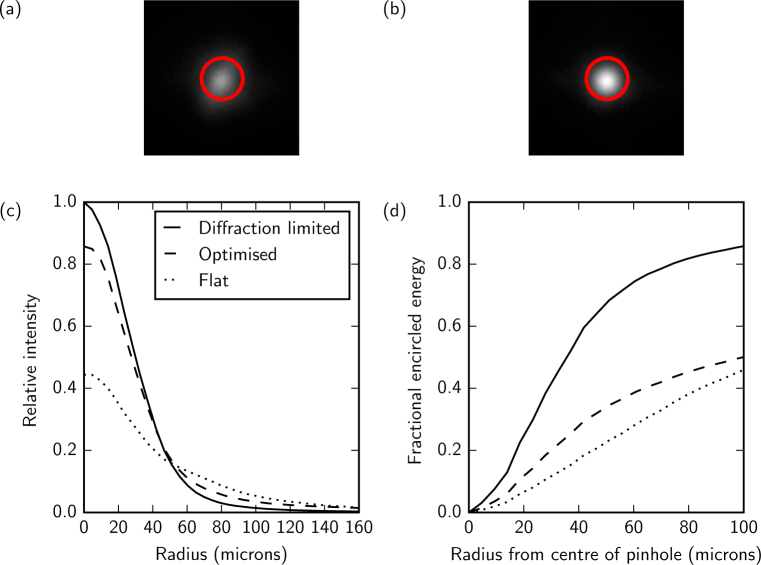
The PSF at the pinhole generated with (a) a flat (zero voltage) DM and (b) the optimum mirror shape determined by maximizing the PSF sharpness. The red circle indicates the Airy diameter for light of wavelength 850 nm for a 7 mm pupil diameter at the eye. The improvement in (c) the PSF and (b) the improvement throughput (encircled energy) are shown and the optimized PSF is compared to that expected from the Zemax model (diffraction-limited).

**Fig. 7:**
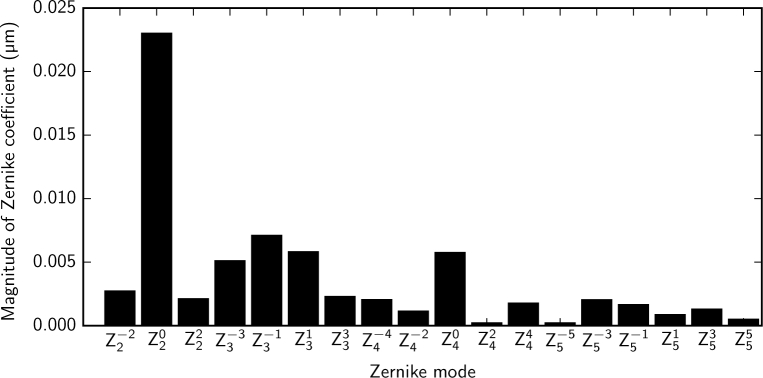
The system aberrations present at the eye pupil, which have been measured using a model eye by comparing the wavefront for a flat (zero-voltage) DM to that when the DM vector has been optimized to maximize the throughput at the confocal pinhole. The dominant aberrations are tip $\left(Z_{1}^{1}=-0.18\mu m\;\text{rms}\right)$, tilt $\left(Z_{1}^{-1}=-0.18\mu m\;\text{rms}\right)$ and focus $\left(Z_{2}^{0}=-0.022\mu m\right)$. Manual compensation for tip and tilt can be achieved by fine-tuning the lateral position of the confocal pinhole. Focus can be compensated manually by fine-tuning the axial position of the confocal pinhole and the focus in the source module. Tip and tilt are not included in the bar chart. The total rms wavefront error excluding tip, tilt and focus is 0.013 *µ*m.

System aberrations are compensated using optimized DM voltages using either the PSF sharpness or the pinhole throughput as a performance metric. The two methods give similar results. We typically use the pinhole throughput as a performance metric, as it uses the APD *in situ* rather than requiring the addition of a fold mirror and camera in front of the pinhole. In this section we refer to an optimization result obtained using the pinhole-throughput metric but we show the resulting PSF detected at the pinhole with a CCD for quantification purposes. The improvement in the PSF resulting from system aberration compensation is shown in Figs. 6(a) and 6(b). Optimization of the PSF produced a 17% increase in throughput for a pinhole diameter equal to one Airy unit (Fig. 6(e)) and used 3% rms of the DM stroke. Assuming that system aberration compensation results in a diffraction-limited PSF, comparison between the WFS measurement with the optimum DM voltages and that with zero voltage applied to the DM gives a measure of the aberrations present in the system. Figure 7 shows that the dominant Zernike modes in the system are tip, tilt and focus. This indicates that the system has a very small wavefront error (0.013*µ*m rms) and that the improvement is mainly achieved by fine-tuning the lateral and axial alignment of the confocal spot to the pinhole. For the measurement given in Fig. 7 the amount of tip $\left(Z_{1}^{1}\right)$ was −0.179 *µ*m rms, which corresponds to a horizontal pinhole alignment error of 28 *µ*m, and the amount of tilt $\left(Z_{1}^{-1}\right)$ was −0.183 *µ*m rms, which corresponds to a vertical pinhole alignment error of 29 *µ*m. The amount of defocus $\left(Z_{2}^{0}\right)$ was −0.022 *µ*m rms, which corresponds to an axial pinhole alignment error of 1.7 mm at the pinhole, which is less than the depth of field of the final lens (9.5 mm). This corresponds to a focus error of 0.01 D at the eye for a pupil diameter of 7 mm. The measured wavefront could be used to fine-tune the lateral and axial position of the confocal pinhole as well as the focus of the source module such that the spot is best-focused on the retina and through the pinhole. This load could then be removed from the DM and a second optimization could be performed to compensate for any remaining aberrations.

### 3.2. Noise reduction

**Fig. 8:**
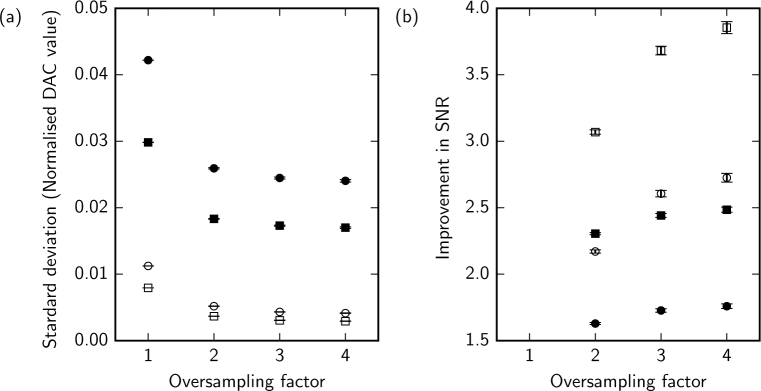
(a) The reduction in noise on a single frame with oversampling, with (filled symbols) and without (open symbols) the additional voltage amplifier. (b) The improvement in SNR with oversampling with (filled symbols) and without (open symbols) voltage amplifier. Error bars represent the standard error of the mean of 30 frames. Single, forward scan frames are represented by circles and the averages of single forward and reverse scan frames are represented by squares. Oversampling by a factor of 4 and averaging the forward and reverse scan frames gives a improvement in SNR of 2.5 in normal imaging conditions (with the voltage amplifier).

Using the digital oscilloscope we oversample the data captured from the detector, allowing single-frame pixel averaging by up to a factor of four, and by a factor of eight when combining the forward and reverse frames. Figure 8 shows the reduction in noise (Fig. 8(a)) associated with oversampling and averaging within a single frame and the improvement in SNR for a typical single AOSLO frame (Fig. 8(b)). Without the voltage amplifier the SNR improved by a factor of 2.7 with an oversampling rate of four and by a factor of 3.8 if the forward and reverse scan images were also averaged. With the voltage amplifier the SNR improved by a factor of 1.8 with an oversampling rate of four and by a factor of 2.5 if the forward and reverse scan images were also averaged. In normal imaging conditions we use the voltage amplifier to keep the incident optical power as low as possible and we oversample by a factor of four and then average the forward and reverse scans. Thus, under normal conditions we expect a factor of 2.5 improvement in SNR. In averaging the forward and reverse scan images we assume that there are no significant differences in the optical distortions between the two scan directions. To validate this assumption we recorded an image of a USAF test chart with the AO loop open, so that the impact of scan-direction-dependent aberrations on image quality can be determined. We used the slanted edge technique [31] to compute the modulation transfer function separately for the forward and reverse scan images and found no difference in image quality.

### 3.3. Image reconstruction

**Fig. 9:**
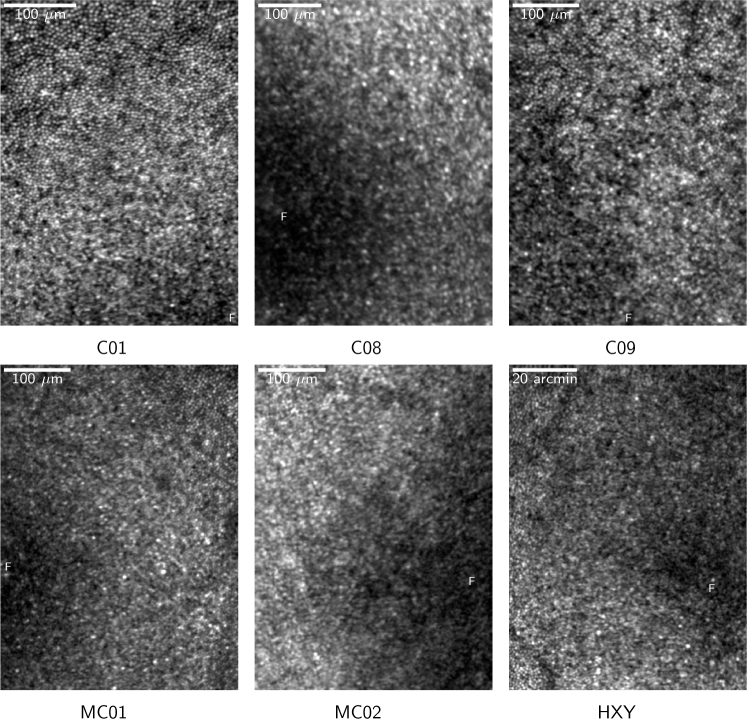
Images near the foveal center of six normal healthy control participants, as described in Table 2. The approximate location of the foveal center is indicated by ’F’.

The system has been used to image the foveal region of six healthy participants, as summarized in Table 2. Participants C01, C08, C09, MC01 and MC02 were recruited as age-matched controls to five patients with Stargardt’s disease (clinical data not presented). These studies were approved by the NHS Research Ethics Committee. Participant HXY was recruited in a separate study approved by the Central University Research Ethics Committee at the University of Oxford. Informed consent was obtained from all participants, and the research adhered to the tenets of the Declaration of Helsinki. Participants’ axial lengths were measured using a Zeiss IOL Master and refraction was measured subjectively. Natural pupil dilation was used and so imaging was conducted in the dark to maximize pupil diameter. Pupil diameter was estimated based on WFS data by running an optimization routine that generated a simulated spot pattern based on a given pupil size. Example images obtained using natural pupil dilation are given in Fig. 9. Raw images (n∼20) were corrected for eye motion using a strip-based correlation technique [14, 15] before being averaged. Sampling resolution in the average image is increased by oversampling individual frames before correcting for eye motion.

**Table 2:** Participant information including spherical (Sph.) and cylindrical (Cyl.) refractive errors, Axial length (AL) measurements obtained with a Zeiss IOL Master, the natural pupil diameter (PD) during imaging estimated from the WFS data (mean and standard error of 30 measurements) and the power measured at the cornea. An axial length was not obtained for participant HXY. C01 was re-imaged with cycloplegia (*).

Participant	Age	Sex	Eye	Sph. (D)	Cyl. (D) Axis (°)	AL (mm)	PD (mm)	Power *µ*W
C01	33	F	OD	0.00	0.00	21.96	6.10 (± 0.01)	85
C01*							6.90 (±0.06)	93
C08	34	F	OS	−3.00	0.00	25.17	6.24 (± 0.11)	120
C09	47	F	OD	−2.25	0.00	23.98	5.15 (± 0.04)	106
MC01	23	F	OD	1.25	−1.00 (174)	22.40	5.64 (± 0.11)	67
MC02	45	M	OD	0.5	−0.25 (90)	23.70	5.50 (± 0.09)	95
HXY	39	M	OD	0.00	0.00	–	6.10 (± 0.08)	77

**Fig. 10:**
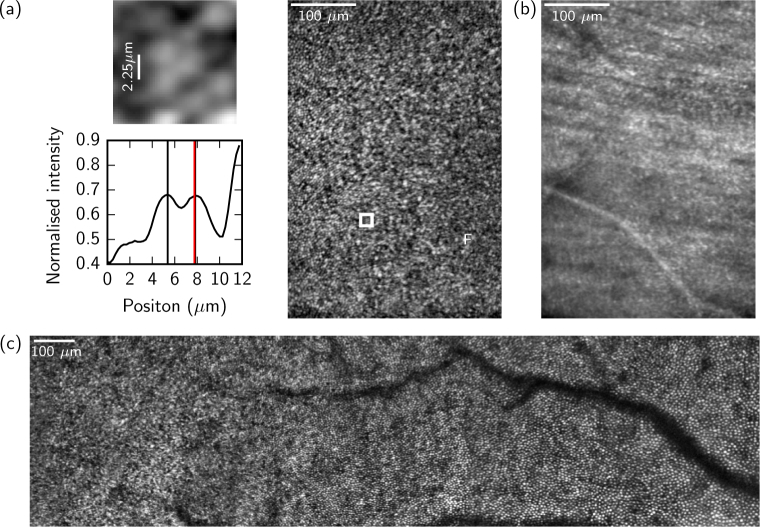
Images from participant C01 (cyclopleged). (a) A 1.6° × 1.0° full frame image of the fovea (approximate location of the foveal center indicated by ’F’). On the left are (top) a 12 arcmin × 12 arcmin patch of the full frame, indicated by the white box, and (bottom) a cross-section through the centers of the two cones to the right of the 2.25 *µ*m scale bar. The 2.25 *µ*m scale bar indicates 1 Airy radius for a 6.9 mm pupil. The center positions of the cones are indicated in the cross-section by the black vertical lines. A 1 Airy radius distance from one cone to its neighbor is indicated by the red vertical line, which overlaps with the center of the neighbor indicating that they are resolved at the diffraction limit. (b) An image of the retinal nerve fiber layer at approximately 5° eccentricity captured by using a trial lens to adjust the focal distance. (c) A montage (generated using Microsoft ICE) of the cone mosaic from the foveal center to 5° eccentricity.

To demonstrate the resolution over a larger pupil diameter, participant C01 was re-imaged using 1% tropicamide, giving a pupil diameter of 6.9 mm. A set of images are given in Fig. 10, showing a 1.6° × 1.0° full-frame image of the fovea, an image of the nerve fiber layer, and a montage of the cone mosaic from the center of the fovea out to 5° eccentricity.

**Fig. 11:**
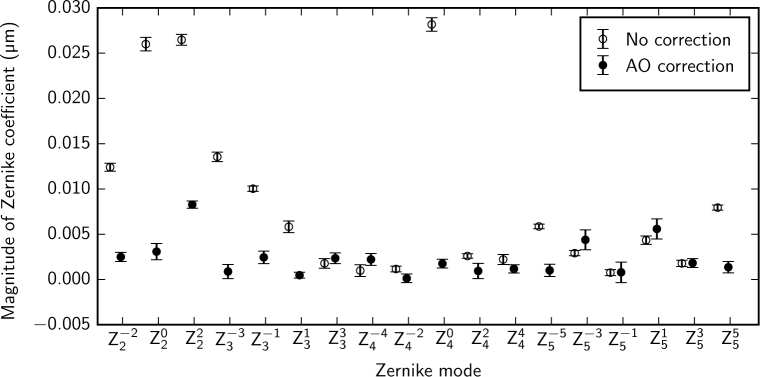
The Zernike coefficient amplitudes (rms *µ*m) calculated by fitting the wavefront with the first 5 radial orders. Data are shown before and after the AO loop was closed, measured over a 6 mm pupil in a participant C01. Natural pupil dilation was used in a dark room and the participant freely accommodated to the focal plane of the AOSLO. The total rms wavefront error reduced from 0.05 *µ*m to 0.01*µ*m. Error bars represent the standard error of the mean of 10 measurements.

**Fig. 12:**
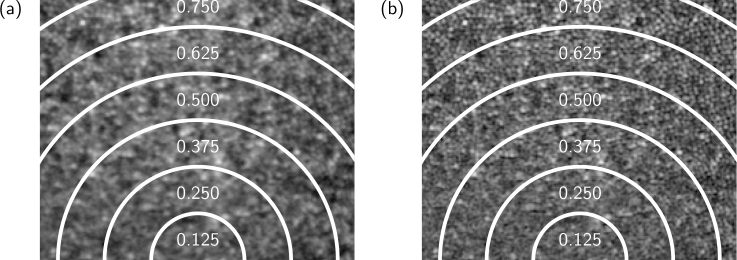
Image of the center of the fovea of a control participant (C01, natural pupil dilation) showing (a) the original image obtained by motion-correction and averaging and (b) the result of deconvolving that image using information from the WFS. An improvement in the contrast between neighboring cones is evident and will aid image-segmentation. Eccentricities in degrees are marked in white. The images are shown on a log scale for clarity.

**Fig. 13:**
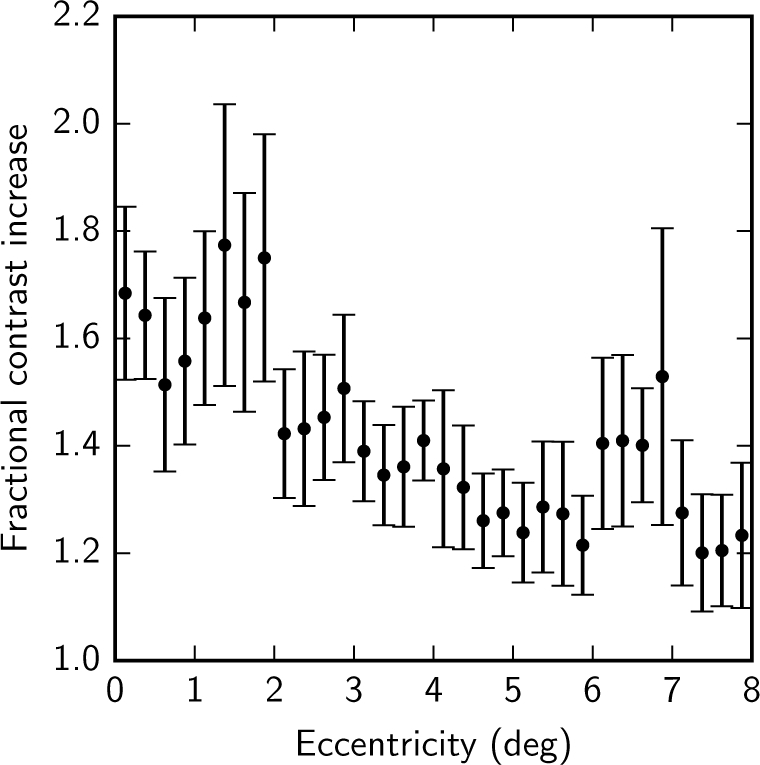
The increase in inter-cone contrast with deconvolution of the image using information from the WFS. The contrast is calculated as the difference in intensity between the dimmest point between a randomly-selected cone and its neighbor, and the centre of that neighboring cone. For each randomly-selected cone the contrast was calculated for each of its six nearest neighbours and averaged. The measurements were made on the images obtained from participant C01 (natural pupil dilation). The average improvement was a factor of 1.4 and the error bars represent the standard error of the mean of the inter-cone contrast measurements of 20 randomly selected cones. We note that cones within the central 0.5° are not well resolved.

Residual aberrations in the system and participant’s eye that are not fully compensated are small (Fig. 11) but can have an impact on image quality, particularly close to the fovea where cone sizes are close to the optical resolution of the system. Using the measurements from the WFS we can additionally use deconvolution [32–34] to improve the inter-cone contrast by compensating for these residual aberrations. Figure 12(a) shows the retinal mosaic at the center of the fovea of participant C01, with natural pupil dilation. Cones are clearly resolved outside of the central 0.5 °; within this diameter some cones are visible but with lower inter-cone contrast and in the center of the fovea poorly resolved cones appear artificially large due to aliasing between the size of the cone and the size of the confocal spot. The improvement achieved via deconvolution is shown in Fig. 12(b). To quantify the improvement, we randomly selected a target cone and calculated the difference between the intensity at the center of one of its nearest neighbors and the darkest point in a path between the center of the target cone and that of the neighbor. Cones were detected in the images using an automatic algorithm that used a Laplacian-of-Gaussians filter followed by a local maximum filter and the results were confirmed by manual inspection. For each randomly selected cone, the inter-cone contrast was taken to be the average of that difference for each of its six nearest neighbours, giving a metric for the ease with which it might be segmented. Figure 13 shows that the inter-cone contrast within the fovea improves by a factor of 1.4 on average when using deconvolution. At all eccentricities the improvement is greater than a factor of 1 (no improvement) by at least one standard error of the mean. The improvement becomes less pronounced with eccentricity, presumably because cones become less densely packed with increasing eccentricity and therefore the contrast between neighboring cone pairs is less affected by residual blur.

## 4. Conclusion

Development of adaptive optics retinal imaging techniques has lead to a range of applications in both clinical and research settings. The AOSLO is informing the characterization of the healthy and diseased retina and building an understanding of the cells responsible for vision. Until now there have been a few major AOSLO design implementations. We have developed a novel AOSLO design that employs off-the-shelf spherical mirrors in an off-axis design based on the Offner relay. This optical design does not suffer from a build-up of astigmatism while keeping the alignment in a single plane and in a compact and modular arrangement. A periscope allows easy viewing of a stimulus display over the top of the AOSLO for visual testing and retinal location targeting. Rather than a frame-grabber or field programmable gate array our system uses an off-the-shelf digital oscilloscope for data capture in combination with custom-written code allowing for greater flexibility and easy customization. We have demonstrated that the system has a very small wavefront error, in line with the diffraction-limited performance predicted from the optical design. Correction for system aberrations, which are mainly a result of lateral misalignments of the confocal pinhole and an axial misalignment causing a focus error, results in a total residual wavefront error of 0.013 *µ*m and increase in pinhole throughput of 17% using only 3% rms of the DM stroke. The customizable data capture method allows us to oversample and average the intensity and position signals, which combined with averaging the forward and reverse scans gives a single pixel average of eight samples, giving an improvement in SNR by a factor of 2.5 under normal imaging conditions.

We have demonstrated diffraction-limited imaging in a healthy participant with the use of cycloplegia. We also have presented example images from six healthy participants showing clearly resolved cones beyond 1° eccentricity and partially resolved cones within the central 1° when using natural pupil dilation and natural accommodation. We have shown that compensating for residual aberrations after AO correction and diffraction through deconvolution can improve the inter-cone contrast by a factor of up to 1.7, which will aid automatic cone-detection and image-segmentation algorithms. With a small footprint, simplified alignment and low-cost hardware interfaces, we hope this new design will aid the transfer of AOSLO imaging to both clinical and research labs for studying visual function in healthy and diseased retina.
